# Application of texture analysis methods for the characterization of cultured meat

**DOI:** 10.1038/s41598-022-07785-1

**Published:** 2022-03-10

**Authors:** Jacobo Paredes, Diego Cortizo-Lacalle, Ane Miren Imaz, Javier Aldazabal, Mercedes Vila

**Affiliations:** 1 Tecnun-University of Navarra, Paseo Manuel Lardizábal 15, 20018 San Sebastián, Spain; 2 Biomedical Engineering Center, University of Navarra, Campus Universitario, 31080 Pamplona, Spain; 3 BioTech Foods S.L., Paseo de Miramón 170, Guipúzcoa, 20014 San Sebastián, Spain

**Keywords:** Techniques and instrumentation, Mechanical engineering

## Abstract

Mechanical characterization supposes a key step in the development of cultured meat to help mimicking the sensorial properties of already existing commercial products based on traditional meat. This work presents two well stablished methods that can help studying cultured meat mechanical characteristics: texture profile analysis (double compression test) and rheology. These techniques provide data about the elastic and viscous behaviour of the samples but also values about other texture characteristics such as springiness, cohesiveness, chewiness and resilience. In this work, we present a comparison of cultured meat-based samples with commercial of the shelf common meat products (sausage, turkey and chicken breast). Results show that both Young’s and Shear modulus in the cultured meat samples can be compared to commercial products in order to understand its properties. The texture characteristics for the cultured meat studied, show values within the range of commercial products. These results demonstrate the applicability of this methodology for the adjustment of mechanical properties of cultured meat products.

## Introduction

Global population is increasing, it is projected to hit 9.77 billion inhabitants by 2050. Demand for animal protein continues to rise due to this population increase and new consumption habits of developing countries, while livestock meat production, the largest protein producer, deals with sustainability concerns (e.g., production of greenhouse emissions, animal welfare, foodborne pathogens…)^1^. All these factors are creating a huge demand for alternative food products that meet customer needs of protein intake.

According to the FAO (Food and Agriculture Organization) in its data per capita protein supply mapped by country, the world population consumes on average 70 g of protein per person per day^2^. For satisfying this high demand of protein worldwide the global meat production keeps on growing, and the FAO estimated that 337.2 million tons of meat were produced in 2021^3^.

The alternative proteins market is therefore seeing a rapid growth. The food industry is searching for alternative protein products while many food innovators are exploring new formulations to impact the existing protein supply chain. Example of these alternatives are plant-based products, insects, and algae proteins. In addition, processing technologies are improving at an incredible pace, such as texturization strategies, intending to mimic the feel and taste of these products, getting closely to traditional meat sensory experience.

Another proposed alternative protein, cultured meat or cultivated meat, the method of producing animal protein by cell culturing in a controlled environment, will help to satisfy these consumers’ demand for animal protein, which will not be sustained by factory farming^4,5^ . This demand, according to United Nations, World Bank and AT Kearny Analysis, will be met by cultured meat (35%), plant-based meat replacement (25%) and conventional meat (40%) by 2040^6^.

Proposed meat alternatives have a wide portfolio of flavours and textures, with different shapes (whole cuts and mince for example), and they can be ingested alone or added to several recipes, pasta, soup, salads… But they all should meet certain criteria for being able to be processed and have appropriateness to be cooked. Moreover, appropriateness can also be influenced by the appearance of the meat substitute plus its flavour and texture. For the development of new foods, characterization techniques should be used to help to simulate and mimic traditional meat texture. These tools could facilitate the integration of meat alternatives into already existing recipes and accelerate customer acceptance by the acquisition of similar innate attributes to traditional products.

In the specific case of cultured meat, to date there is no experimental description of its mechanical properties and/or textural behaviour. The only information available describes the theoretical changes that could be expected based on its nature of production^7^. These sensorial properties are derived from the molecular characteristics of the product, and as cultured meat is still on its infancy, the study and understanding of its properties is of outmost importance to create knowledge on the elaboration of this new alternative protein into products.

Part of the fundamentals properties of food intended for oral consumption are based on its mechanical performance when masticated, and on the rheology of the matrix to be formed into a bolus^8^. Therefore, among the several available texture methods for the characterization of properties specific to meat, herein we propose the use of the texture profile analysis (TPA) for determining cultured meat properties^9,10^. Texture profile analysis is based on a double mechanical compression test to provide insight into how samples behave when chewed. The main advantage of TPA is that many parameters can be obtained with a double compression cycle.

For example, hardness, springiness, cohesiveness, chewiness, etc., and combinations of them could give rise to information of others, for example, hardness, springiness, and cohesiveness altogether allow the calculation of chewiness. But the textural character of meat is not only given by understanding a singular attribute such as hardness, springiness or cohesiveness, the texture is also connected with consumers' sensory feelings expected when tasting that food type^11^. The Warner–Bratzler apparatus was also used for mechanical characterization of food samples. It consists of a V- notched “blade” that exert a shear cutting movement over a cylinder of the sample. In contrast with the double compression test (TPA), this technique simulates best the cutting effect rather than the chewing. Even though these are two accepted and valid methods for mechanical assessment of food, Novaković stated that TPA is more suitable for raw meat^12^.

Rheology is specially indicated for viscoelastic material characterization; it provides the shear behaviour of the sample which is complementary to the TPA characterization. First rheological studies of meat and sausages dates from the 70’s, as well as the application of this technique to other foods like dairy products^13^. These data can be used to understand the viscous behaviour of the material. Even more, flow behaviour is critical for food processing, for instance for extrusion processes^14^. In this sense, cultured meat has awakened great interest for meat processing, as it allows the possibility of replicate not only the texture properties but also the appearance of the meat^15,16^. Rheological characterization would provide the necessary information to control both the manufacturing process and the final product characteristics.

TPA combined with rheological tests, can quantify multiple textural parameters and, being a reproducible instrumental approach, it less time consuming and costly to conduct when compared with a sensory panel texture evaluation. Herein, we present a comparative study of textural and rheological properties of different types of meat versus a sausage made with cultured meat, proving that is a useful method to better understand the characteristics of cultured meat.

## Materials and methods

### Sample preparation

Different meat samples were selected for a comprehensive comparison of mechanical properties between natural, processed, and cultured meat: (1) commercial processed Frankfurt-style sausages (sausage), (2) processed turkey breast cold cut (turkey), (3) non-processed raw breast chicken (NP Chicken) and (4) Frankfurt-style sausage made of cultured meat (BTF-CM). All samples were purchased from local Spanish markets. Cultured meat was provided by Biotech foods S. L. (San Sebastian, Spain). None of these products were frozen and they were kept at 4 ºC. During storage, special precautions were taken to ensure that the samples did not lose their original moisture content, especially for the non-processed chicken. Before testing, they were taken out of the fridge and kept at room temperature for 1 h.

**Figure 1:**
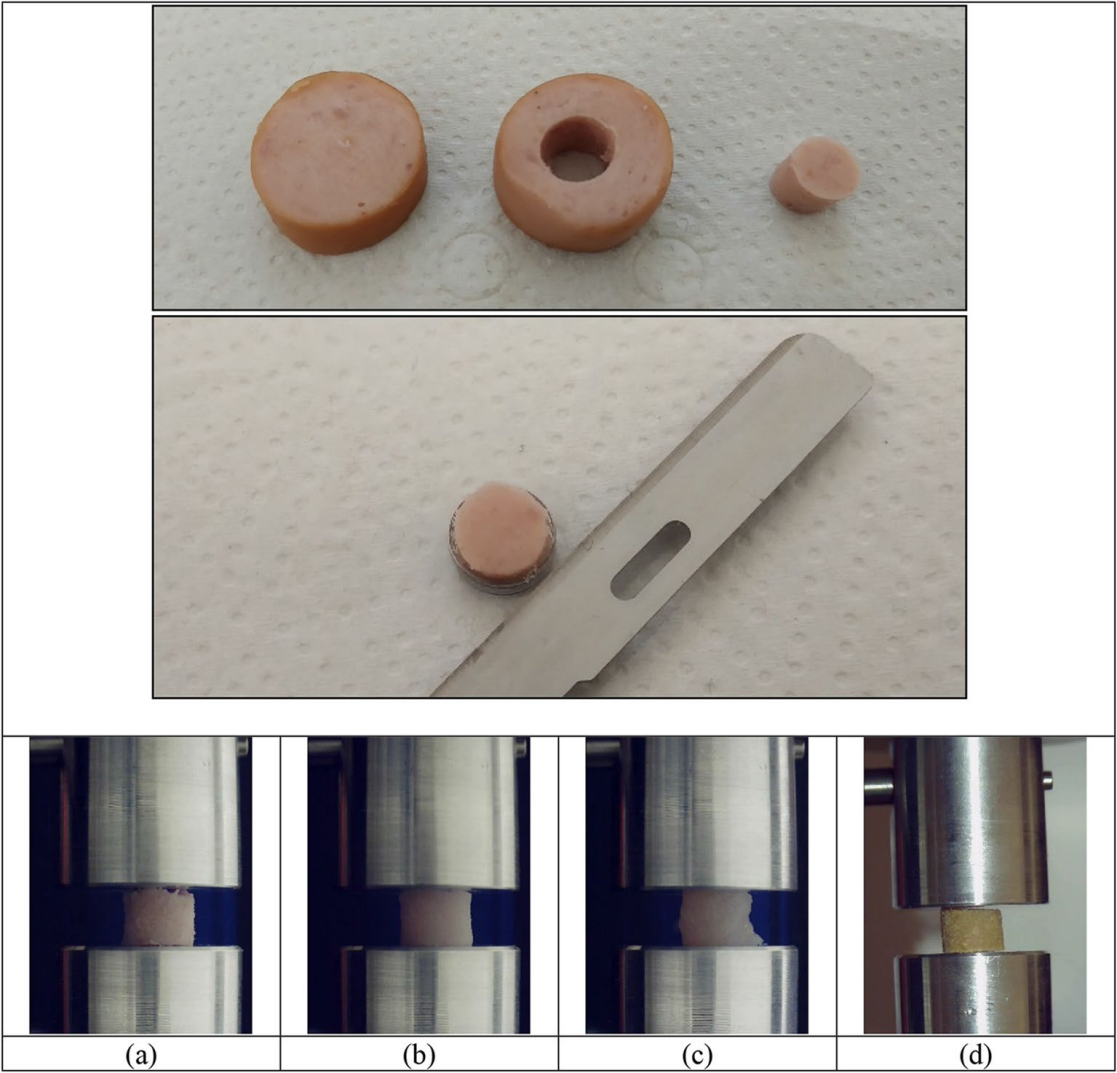
(Top) Process for obtaining the samples. Obtaining a cylinder using an 8 mm punch and placing it on the apparatus to fix its thickness by cutting it to the desired thickness. Process example shown correspond to a sausage. (Bottom) Images correspond to tested samples of Frankfurt style sausage **(a)**, processed turkey **(b)**, non-processed chicken **(c)** and cultured meat Frankfurt style sausage **(d)**.

Samples were cut into cylindrical probes, first shaped with an 8 mm punch, as shown in Fig. 1 top. Then samples were cut to the desired thickness using a methacrylate plate template with the same thickness as the final sample and with a cylindrical hole with the diameter of the final probe: the cylindrical piece of material to be tested was inserted into the hole in the plate and the thickness of the sample was reduced by sliding a microtome blade over it (Fig. 1 centre). The sample part under the blade had the same thickness as the plate.

The process of manufacturing samples was easier and more consistent with processed food. Chicken breast presented some difficulties due to fibre orientation and moisture. For this product, only uniform and continuous areas were considered for sampling; edges, fat and other imperfections were immediate discarded. Figure 1 bottom shows images corresponding to examples of tested samples for all products compared in this work.

For each material, at least six samples were studied using TPA and five samples were used for rheology experiments.

### Texture profile analysis (compressive tests)

**Figure 2:**
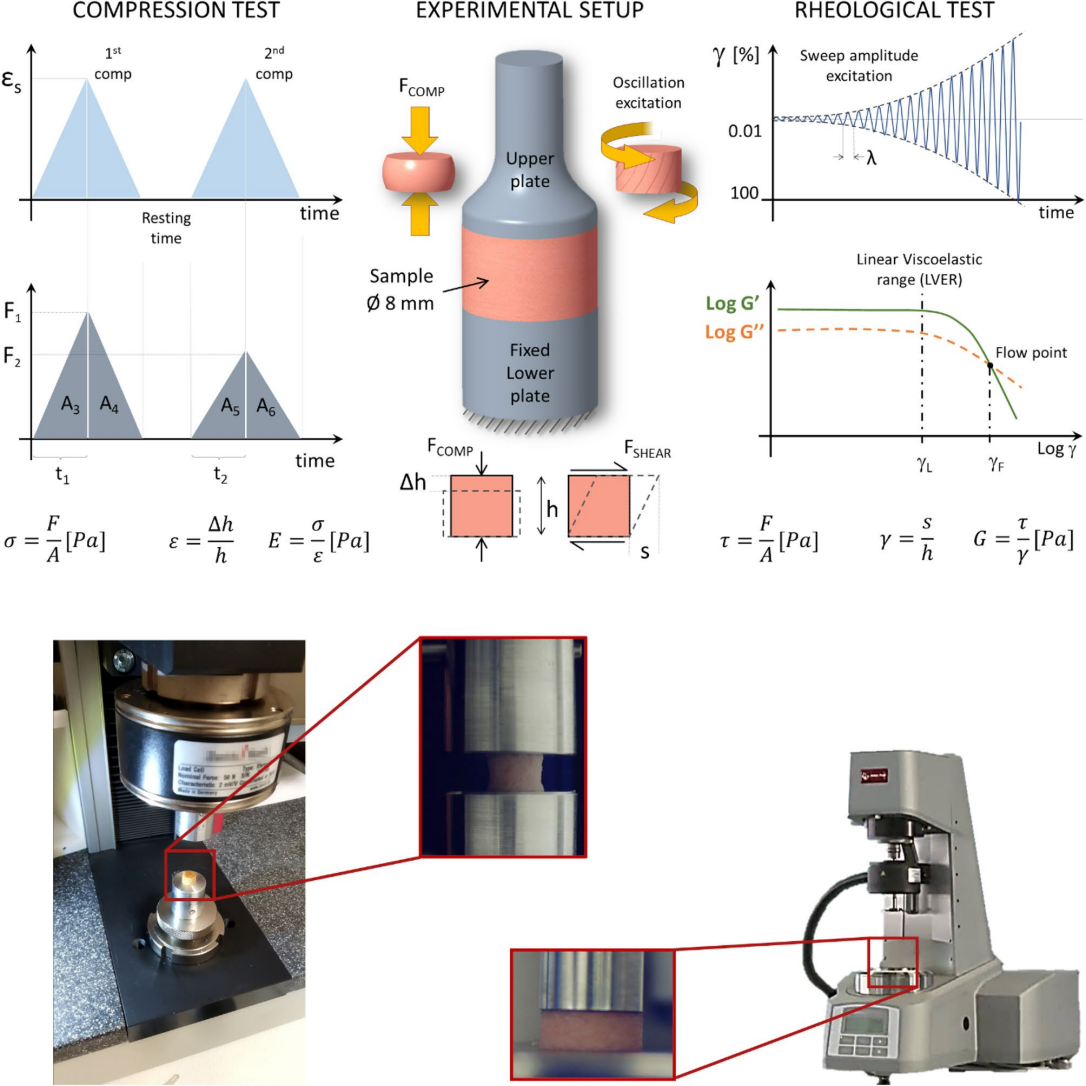
(Top) Schematic representation of both tests: texture profile analysis and rheology. Experimental setup consisting of two parallel plates, which holds the sample (with the same diameter (8 mm) for rheology). Excitation profiles and theoretical sample response (key parameters) are shown. (Bottom) Representative images of used setups for TPA (left) and rheology (right). The images chosen are of Frankfurt style sausages.

The Texture Profile Analysis (TPA) test, as already mentioned, consists of applying two cycles of deformation to a sample of the material to be studied. Between these two compression cycles, the sample rests for a certain time. The first column of Fig. 2 shows a diagram of what happens during the TPA test. The upper part shows the applied deformation cycle, and the middle part shows the force with which the material responds. In this second diagram, it can be seen that the force with which the material responds is lower in the second cycle. The mathematical expressions in the lower part of the figure correspond to (1) the mechanical stress suffered by the material as a function of the force applied and the cross-sectional of the sample, (2) the dimensionless strain of material as a function of the initial height of the sample and the shrinkage produced at an instant of the test and finally (3) the elastic modulus, or also called Young's modulus, as the ratio between stress and strain.

The analysis of materials response allows numerous parameters to be obtained, but in this work the following will be considered:

- Young's modulus is obtained fitting the linear part of the force–displacement applied by the specimen with a linear equation. The slope of this equation corresponds to the Young's modulus or stiffness. Stiff materials have high moduli and vice versa.
- Hardness is obtained by observing the maximum load reached during the first deformation cycle (F_1_). It is related to the stiffness of the material.
- Cohesiveness corresponds to the ratio between the area under the time/force curve during the second cycle (A_5_ + A_6_) divided by the area during the first cycle (A_3_ + A_4_). This parameter is related to the consistency of the material. If the material withstands the first cycle without disintegrating, the value will be close to 1, but if it disintegrates completely, it will be close to zero.
- Springiness corresponds to the ratio between the time needed for the material to reach the maximum load since it starts to deform in the second cycle (t_2_) and the time needed for the first cycle (t_1_). This parameter is related to the recovery of the material and its viscoelastic properties.
- Chewiness is a parameter obtained multiplying the hardness times the cohesiveness time the springiness. It is related with how easy a material can be bitten.
- Resilience is calculated by dividing the upstroke area (A_3_) by the downstroke area (A_4_) of the first compression cycle. It is related with the plastic deformation of the material. If material does not deform plastically its value will be one but it the material do not recover its shape after the first compression cycle its value will increase.

TPA was performed on a universal uniaxial testing machine (ZwickiLine Z1.0, ZwickRoell GmbH & Co. KG, Ulm, Germany). Figure 2 (bottom) shows the experimental setup used for the TPA on the left. For measuring the force produced by samples, the load cell used was a 50 N Zwick/Roell Xforce P. The software for controlling the machine and recording data was the Zwich/Roell testXpert III v1.4. For determining the initial contact between compression plates and sample a 0.01 N load threshold was used, i.e., when a load of 0.01 N is detected the test automatically starts. During load, machine crosshead moves at 3 mm/s downwards compressing the sample until a deformation of 0.5 is reached, i.e., a 50% of its original length.

This loading process takes less than one second. This time is like the typical period of a standard bite^17^. When the maximum deformation is reached, the movement is inverted, keeping the speed, to the original position. This cycle is followed by a pause of 1 s and the second cycle repeats the pattern of the first.

### Rheological characterization

The rheological analysis consists of applying shear stress to a cylindrical sample placed between two parallel plates. The lower plate is fixed, but the upper plate rotates while the instrument can measure the sample response to the shear deformation. Commonly the deformation is set to specific values while the instrument measures the torque applied to achieve such displacements. These values provide the shear strain of the material for a range of speeds and deformations. The sample and the plates should have the same diameter ensuring proper contact between them.

The rheological characterization was performed on a rheometer (MCR 301, Anton Paar GmbH) equipped with parallel plates (diameter 8 mm, gap 3 ± 0.1 mm). Amplitude sweep tests (0.01–100% Gamma, 10 Hz) were performed on the freshly prepared samples. This test provides information about the complex shear modulus (G*) given the two main parameters: storage modulus (G’) and loss modulus (G”). These parameters (as Fig. 2 bottom shows the experimental setup used for the rheological characterization on the right side. a function of the deformation amplitude) allow the analysis of elastic and viscous behaviour of the sample under shear stress.

Figure 2 also shows the experimental setup of rheological tests: the oscillatory shear excitation and the response of the sample as a function of the amplitude deformation (γ). Frequency sweep tests (in the LVER, 0.1%, 0.1 to 100 rad/s) were discarded due to the dominant elastic behaviour of the samples (the lack of fluidity, and the low viscosity). Even though this test is mandatory for shear-thinning or shear-thickening analysis^18^, the materials studied in this work behave just as elastic solids. The Linear Viscoelastic Range (LVER) was determined as the range of the deformation (the maximum γ) from which G’ present a relative variation as maximum as 5%. The ISO 6721-10 was taken as a reference, although other standards use a maximum relative variation of 10%. All tests were performed at room temperature and recorded for the later analysis. No normal force was applied to the specimens as it induced artifacts at low strain (data not provided): the minimum compression force was used to ensure an adequate adhesion of the sample to both plates.

Different thicknesses were tested prior to sample comparison analysis to evaluate sample performance under shear conditions, adherence, wall effect, slippage, etc. These effects are critical in the case of viscoelastic fluids, but not that much on elastic or viscoelastic solid materials. Usually for viscoelastic liquids (gels, creams, pastes, etc.) the recommended thickness is the range of 0.5 to 1 mm. However, according to previous studies^19,20^, too small sample thickness could lead to artifacts and undesired effects. Moreover, in this study 1, 3 and 5 mm thick samples were tested observing no significant differences in their performance (data not provided). Preparing thin and uniform meat samples consistently is very challenging and therefore 3 mm thickness was selected for all samples. During the sweep amplitude oscillatory test loss of adherence was observed at high deformations (~ < 10%) on all the samples.

### Statistical analysis

RStudio^21^ was used to perform the statistical analysis. Analysis of variance (ANOVA) was first performed to evaluate the complete set of the data. These results show if there is at least one group significantly different from the others, but not specifically which one. This data provides an insight on the range of all types of meat.

Finally, a t-student is preferred to provide statistical significance when comparing the cultured meat to specific meat groups (with the same characteristics), as in this work. In our case, we performed a comparison between the cultured meat sample and the three different reference types of meats to analyse their differences. P values below 0.01 were considered significant.

## Results and discussion

### Texture profile analysis

**Figure 3:**
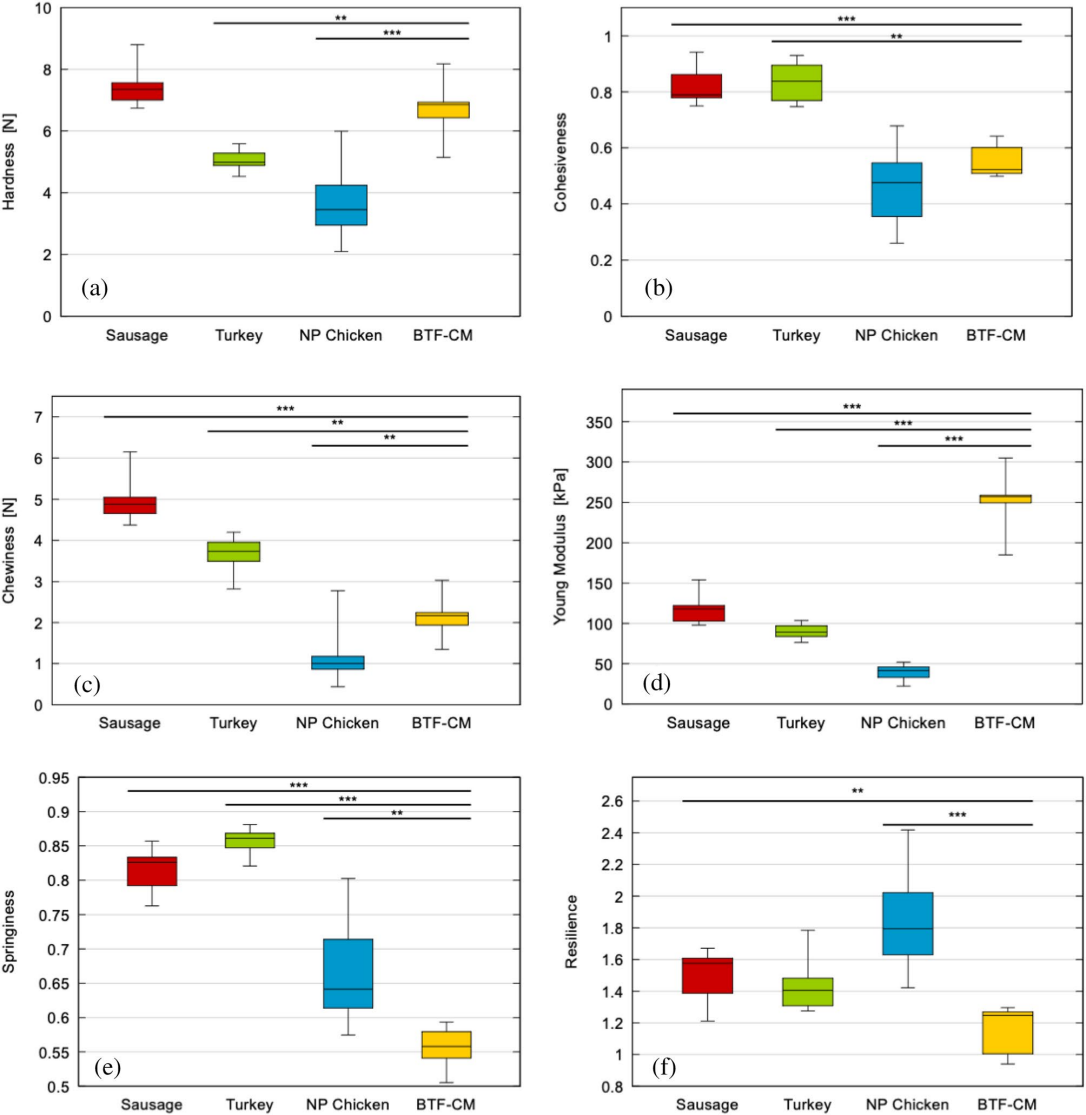
Box plot of hardness **(a)**, cohesiveness **(b)**, chewiness **(c)**, Young’s modulus **(d)**, springiness **(e)**, and resilience **(f)** of the three traditional meat references and the cultured meat (p values < 0.01 *; < 0.001 **; < 0.0001 ***).

Figure 3 shows box plot of all previously mentioned parameters obtained from the TPA tests (N ≥ 6). The statistical analysis of these data concluded that, for all these six parameters, the ANOVA analysis for the complete set of data was very significant (p < 0.0001). This reveals that at least one of the groups was at a different range than the other products. The comparison of the cultured meat with each commercial group was also significant for most of the considered properties and meat types (marked in the figure as p values < 0.01 *; < 0.001 **; < 0.0001 ***).

Analysing the hardness related to the first bite from the values represented in Fig. 3a, the Frankfurt sausages, both, the commercial and the cultured meat prepared one, show similar values as statistically it cannot be said that there is a difference between them. As expected, these values significantly differ from those of cold cut turkey and fresh chicken. It is worth to notice that in Frankfurt sausages, all the fibrillar and macromolecular components of meat are broken apart eliminating all the innate textural properties of the initial meat. The hardness parameter could be associated to the first bite feeling, so it is critical from the perspective of the final consumer.

The cohesiveness analysis showed that unprocessed chicken presents the lowest value. This could be explained because this non-homogenous and fibrous material partially degenerates during the experiment. Likewise, the cultured meat Frankfurt sausage also presents a relatively low value, similar to the chicken, due to a possible disaggregation during the test. This fact could explain why, even though Frankfurt sausage made of cultured meat seems to be a more rigid material, its hardness is not much higher than that of the other materials considered. Commercial processed products, such the cohesiveness analysis showed that unprocessed chicken presented the lowest value. This could be explained as sausages and turkey, show values close to 1, which indicates that they do not disaggregate during the tests and that they are more elastic.

Considering the values obtained for chewiness, the cultured meat Frankfurt sausage presents values between those of processed turkey and unprocessed chicken meat. This indicates that the chewiness is within the range of the other studied materials.

The Young’s Modulus analysis was the parameter that showed the larger differences between the studied cultured meat Frankfurt sausage and the traditional meat products. Commercial Frankfurt sausage shows higher values of stiffness than turkey, and turkey is stiffer than unprocessed chicken, but the values show similar order of magnitude moduli. However, cultured meat prepared Frankfurt sausage, presents a significant higher value than the commercial sausage suggesting that the process to prepare it gives rise to a stiffer product. Even though is not easy to associate this engineering parameter to a sensorial feeling, this is a very useful way for understanding what possible modifications could be done on the meat processing strategies to obtain similar values than those already accepted by the consumers.

The springiness value gives an idea of how long it takes for the material to recover its shape and properties after compression. The value of this parameter for Frankfurt sausage prepared with cultured meat is lower than the commercial one and the processed turkey, but it is in a similar range of the fresh chicken (0.61 vs. 0.54), which indicates that it is more sensitive to the speed of deformation and therefore is apparently more viscous-like than the others.

The resilience of the studied materials is comparable in all cases except for unprocessed chicken meat, which shows significant higher values. This indicates that chicken meat deforms permanently after the first loading cycle. This is due to its inhomogeneous nature which in many cases results in delamination of samples.

It is worth to notice that the dispersion in the results has generally lower values in the case of the processed turkey. One possible reason is the high degree of homogeneity of the material. On the contrary, the highest dispersions were obtained for unprocessed chicken meat. This dispersion is due to the heterogeneity of the unprocessed material. The sausage and cultured meat samples have shown intermediate dispersions in general.

Cultured meat’s organoleptic properties were anticipated to be different from traditional meat. Traditional meat derives from a complex muscular tissue formed mainly by muscular fibres 90% (fibre bundles, myofibrils…), connective tissue 10% (endo-, peri- and epimysium), and to a lesser extent by fat tissue, vascular and nervous tissues. The transition from muscle to meat happens during the post-mortem stage and the successive maturation under a fine control of different parameters such as time, temperature, pH, stress,… During this process meat acquires a unique series of characteristics in terms of flavour, colour, taste and texture^7^. Cultured meat is still mainly obtained from a muscle tissue production by cells, and its organoleptic development after the cell culture is under study. Future data on its time, temperature and pH dependency transformation would be of high importance to complement the findings shown here.

It is difficult to make a comparative analysis of this dataset with previous works as to date there is no data available for cultured meat, and when referring our values to other traditional meat studies the literature presents a high dispersion of values referred to different sample origins, sample preparation, cooked vs non cooked, storage, etc.…

Most of the studies use the texture analysis to evaluate the effect of additives or different formulations on the final product, or to evaluate the differences between uncooked or cooked samples. Nevertheless, there are some interesting observations that could be done comparing the mechanical properties of the presented data with the published data.

The cohesiveness and the springiness of uncooked unprocessed chicken presents comparable values to those reported in bibliography^22,23^. Data found in literature is consistent with the sausage comparison, the hardness parameter is higher after the meat is cooked, and the cohesiveness and springiness decrease^10,24^.

### Rheological characterization

**Figure 4:**
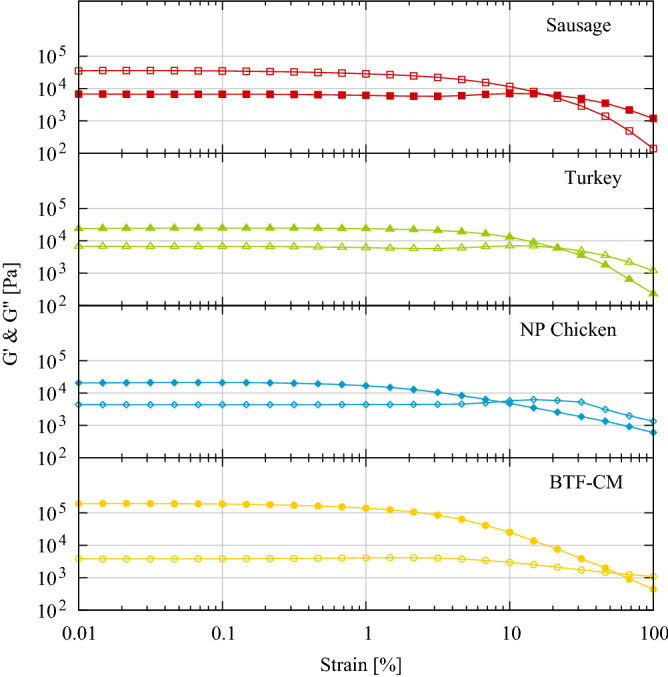
Mean values (N ≥ 4) of storage modulus (G’) and the loss modulus (G”) from sweep amplitude test for meat samples (γ = 0.01–100%, frequency = 10 Hz, gap 3 mm).

Figure 4 represents the mean value (N ≥ 5) of the storage modulus (G’) and the loss modulus (G”) as a function of the deformation amplitude in logarithmic scale. All samples exhibit a similar behaviour where G’ is significantly higher than G” for deformations below approximately 5%. All samples present an initial plateau for both parameters until dropping their values. However, this effect is caused by the slippage of the sample at the lower plate, not due to change of behaviour from solid to liquid. Even if the graphs show a flow point (where the curves of G’ and G’’ cross over) the samples were conserved intact. Assuming these limitations, the calculated LVER for the samples is 0.14, 0.33, 1.5 and 0.15% respectively. Even though there is not much information of this type of analysis in the bibliography, another similar study with meat analogues showed the same behaviour of the samples^25^.

**Figure 5:**
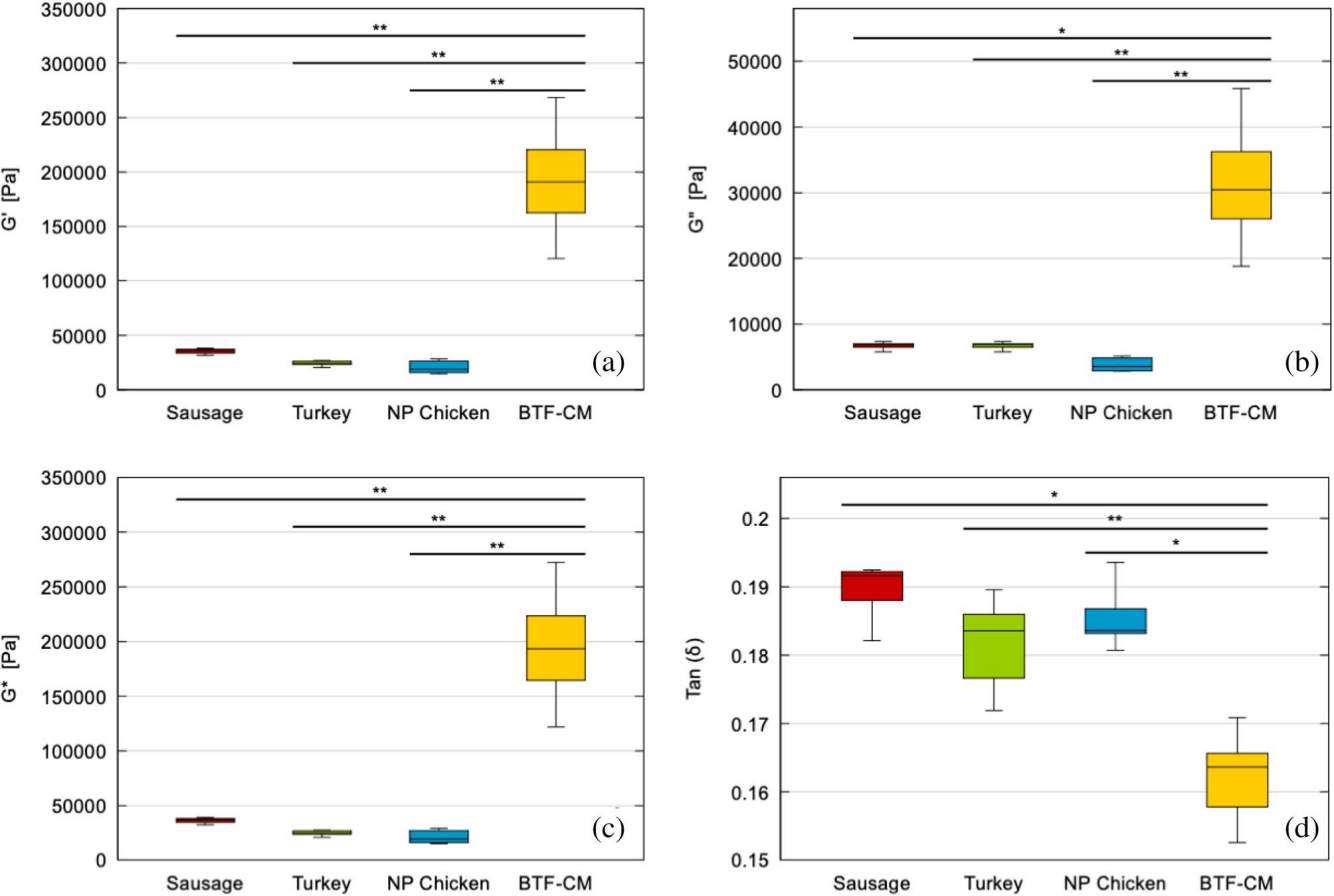
Box plot of **(a)** storage modulus (G’), **(b)** loss modulus (G”), **(c)** complex shear modulus G* and **(d)** tan (δ) of the three meat references and the cultured meat (p values < 0.01 *; < 0.001 **; < 0.0001 ***).

Figure 5 shows box plot of storage modulus (G’), loss modulus (G”), complex shear modulus (|G*|) and the ratio between G”/G’ as tan (δ) within the LVER. These values were calculated from each measurement as the average of the plateau values of both parameters G’ and G”. G* and tan(δ) were then calculated accordingly (N ≥ 5).

$${G}^{{{\prime}}}=\frac{\sum_{{\gamma }_{0}}^{{\gamma }_{L}}{{G}^{{{\prime}}}}_{\gamma }}{{n}_{\gamma }}$$

$${G}^{"}=\frac{\sum_{{\gamma }_{0}}^{{\gamma }_{L}}{{G}^{"}}_{\gamma }}{{n}_{\gamma }}$$;

where γ_L_, deformation limit for the LVER, n_γ_ = number of measurements in the LVER.

$$\left|{G}^{*}\right|=\sqrt{ {G}^{{\prime}^2} +{G}^{{\prime\prime}^ 2}}$$

$$\mathrm{tan}\left(\delta \right)=\frac{{G}^{"}}{{G}^{{{\prime}}}}$$

The statistical analysis of these data concluded that, for all these four parameters, the ANOVA analysis for the complete set of data was very significance (p < 0.0001). Again, this fact reveals that at least one of the groups was at a different range than the other products.

The comparison of the cultured meat sausage with each commercial group was also significant for each parameter (marked in the figure as p values < 0.01 *; < 0.001 **; < 0.0001 ***).

From the rheological analysis, one can see that values and trends from sweep amplitude tests confirm that samples present an elastic behaviour and consequently can be described as solid viscoelastic materials. The storage modulus is much higher than the loss modulus for deformations in the LVER and therefore the ratio G”/G’ (tan(δ)) is very low, in the range of 0.15 to 0.2. Despite the differences in their origin or their preparation process, the mechanical behaviour of the samples is quite similar. Specifically, the commercial and cultured meat Frankfurt sausages are made of ground meat and cooked, the processed turkey breast partially maintains the fibre structure and orientation (it is a fusion of multiple breast pieces) and is also cooked, the chicken breast is not processed and raw. All samples slip from the lower plate before breaking the internal structure or flow. This effect occurred for all rheological measurements regardless thickness. It means that the internal structure is stronger than the adhesion force to the plates. High roughness plates could be used instead of the standard sandblasted ensuring a better adherence and a later slippage, but it will not provide any extra information. The application of normal force should be avoided to increase adhesion of the sample, as it introduces a complex deformation on the sample hindering an accurate shear analysis.

As these samples present a strong elastic behaviour it is not useful to further analysis other rheological parameters or the study of how the deformation speed affects the mechanical behaviour of the samples (frequency sweep test). In our case, one can expect to have no changes in the shear modulus with any variations of the experimental conditions. In fact, the samples exhibit constant uniform horizontal G’ and G” values for the range of the frequency analysed.

The results of Fig. 5 confirmed that the cultured meat prepared sausage presents the highest shear modulus represented as |G*|, as well as the highest G’ and G”. In addition, the commercial sausage presents the highest shear values of the commercial samples while the raw chicken the lowest. The water content can explain this behaviour as diminish the effect of the fibres of the breast muscle. The fact that the slippage of the sample occurred at lower deformation in the chicken than in the rest also supports this hypothesis.

Results corresponding to the tan(δ) show a different perspective of the comparison of the mechanical behaviour of the samples. Cultured meat prepared sausage presents the lowest ratio while the other samples are similar in values and deviation. The lower the tan(δ) the higher the difference between elastic and viscous behaviour, i.e., the more elastic the material and the less viscous. However, all samples present the same order of magnitude, meaning that the texture characteristic regarding the shear analysis is very similar in all of them.

These results are consistent with the data from the TPA, where the analysis suggests that regardless the type of applied strain, the behaviour of the materials is more elastic than viscous. On the other hand, the cultured meat prepared sausage proved to be stiffer than the other commercial meats. However, the other sensorial parameters are within the range of the other types of meat, which is as important for customers’ acceptance as the classical mechanical modulus (E and G).

## Conclusions

This work presents the results of the mechanical characterization of cultured meat products and their comparison with commercially available meat products. This study used two complementary techniques: Texture Profile Analysis and Rheology. The first one is a two-consecutive compression test that compare material response between both test and the second one analyses the viscoelastic properties of the material. In addition, this work also presents a statistical analysis suitable for a comparison of different types of meat samples.

The analysis of these four specific samples shows that the cultured meat product exhibits similar texture characteristics for parameters such as hardness, cohesiveness, springiness, chewiness and resilience compared to commercial meats and higher elastic and shear modulus (E and G). Results from the TPA are consistent with rheological experiments. Considering the elastic dominant behaviour of the samples, TPA seems to provide more valuable information than the rheological characterization.

This methodology has proven to provide valuable information for the development and optimization of cultured meat product processing strategies and has helped to unveil some of the unknown parameters in such an incipient field. In a quantitative and rapid manner, using the proposed methods, researchers can adjust different compositions, additives, or process parameters to mimic mechanical texture properties of meat products that are already accepted by customers.
